# Molecular markers relevant to myocardial injury following dental extraction in patients with and without coronary artery disease

**DOI:** 10.1038/s41405-019-0018-8

**Published:** 2019-07-09

**Authors:** K. M. Habbab, F. D’Aiuto, M. A. Habbab, S. R. Porter

**Affiliations:** 1 UCL Eastman Dental Institute, London, England UK; 2 Prince Sultan Cardiac Center, Riyadh, Saudi Arabia

**Keywords:** Special care dentistry, Periodontitis

## Abstract

**Objectives:**

The aim of this study was to characterize biological changes following dental extractions in patients with and without coronary artery disease (CAD).

**Materials and methods:**

Forty-five patients (36 males and 9 females) referred for dental extraction underwent treatment and provided blood samples before, immediately after, and 24 h after the procedure. A broad array of biomarkers was employed to assess myocardial injury (highly sensitive troponin T, hs-TnT), bacterial burden (LPS endotoxin activity), and systemic inflammation (CRP, fibrinogen, IFN-γ, IL-1β, IL-6, IL-8, IL-10, IL-12, and TNF-α).

**Results:**

Dental extraction in patients with and without CAD was associated with rises in hs-TnT (*p* = 0.013), hs-CRP (*p* < 0.001), fibrinogen (*p* = 0.005), endotoxin activity (*p* < 0.001), IFN-γ (*p* < 0.001), IL-6 (*p* < 0.001), IL-8 (*p* = 0.011), and IL-12 (*p* < 0.001) at 24 h compared with immediately post procedure. Changes in systemic inflammation and endotoxin activity were more evident in those with hs-TnT rise.

**Conclusions:**

Simple dental extractions may cause mild increase in hs-TnT, indicating minor myocardial injury in both patients with and without CAD. Acute systemic inflammation and endotoxemia could represent a possible link between invasive dental treatment and increased risk of acute cardiovascular events. These findings indicate that invasive dental treatment (as simple as a single dental extraction) may impact negatively on clinical outcomes in dental patients, especially those with CAD.

## Introduction

Inflammation plays a major role in the pathogenesis of atherosclerosis and its complications.^1^ Peri-procedural minor myocardial injury related to systemic inflammation following coronary interventions can worsen the clinical outcome of patients with coronary artery disease (CAD).^2,3^

Poor oral health, especially in the form of periodontitis, is known to cause an elevation of inflammatory biomarkers, and its treatment has been linked with both acute and chronic changes in systemic inflammation in otherwise healthy individuals.^4^ A recent case series reported an increased risk of mortality, due to vascular events in patients with CAD following dental treatment.^5^ Epidemiologic evidence links invasive dental procedures with an increased risk of vascular events, but the mechanisms that underlie these trends remain poorly understood.^6^ The acute influence of invasive dental procedures, such as exodontia upon CAD, and the possible mechanisms involved, have not been described in great detail.

Invasive periodontal treatment (nonsurgical and surgical therapy) as well as the third molar extraction cause increases in circulating markers of inflammation, coagulation, and endothelial cell activation in 7 days following treatment.^7–9^ These acute changes are also associated with a transient state of vascular dysfunction and pro-coagulant state,^8^ but have yet to be linked to increased mortality or clinical events in patients (healthy or with CAD). Bacteremia following exodontia and other dental treatments^10^ may cause myocardial ischemia by a variety of possible mechanisms.^11–19^ In addition, tissue injury caused by local surgical trauma is known to trigger a systemic inflammatory and pro-coagulant state.^20–22^

All of the above mechanisms might add to the inflammatory burden of patients with CAD and have a direct influence on vulnerable coronary atherosclerotic plaques, leading to plaque fissuring/rupture, thrombogenesis, and/or microembolization within the myocardium, leading to microinfarcts and minor myocardial injury.^23,24^

The aim of this study was to characterize biomarker changes relevant to myocardial injury, following invasive dental procedures (predominantly dental extractions) in patients with and without coronary artery disease. Changes in circulating biomarkers of myocardial ischemia, endotoxemia, and systemic inflammation were determined before and after the invasive dental procedures.

## Materials and methods

### Study design and patient population

In this prospective clinical study, we enrolled forty-five consecutive adult patients (36 men and 9 women) referred to the Special Care Dentistry Unit of the University College Hospital NHS Foundation Trust (UCLHT) Eastman Dental Hospital or UCLHT Heart Hospital for routine pre-cardiac surgery dental assessment/treatment. Participants were categorized into two groups, patients with and patients without CAD, by history and the available results of coronary angiography, echocardiography, or ECG. All patients in the CAD group had evidence of CAD by coronary angiography. Eligible participants were excluded if immunocompromised (e.g., organ transplant or taking immunosuppressants), had episodes of acute inflammation, or had a diagnosed chronic immune or inflammatory disease (assessed by the examining cardiologist). All patients gave informed consent and the study was approved by the National Research Ethics Service (reference number, 10/H0715/42).

### Dental examination and therapy

After a baseline visit and collection of a complete medical and dental history (assessed by interview), dental status (i.e., decayed, missing, and filled teeth), oral mucosal health, and clinical periodontal parameters were recorded by a single trained clinician. All participants needed dental extractions. A single operator performed all dental treatment. A topical analgesic gel (lidocaine 2%) was applied over the site of injection of the local anesthetic. Local anesthetic (1.8 ml of articaine HCl 4% with 1:1,00,000 epinephrine carpule) was injected into the site of the tooth (or teeth) of concern. The number of carpules used was determined on a case-by-case basis according to the site of the teeth in concern, the number of the teeth, and the patient’s perception to pain. According to the current National Institute for Health and Care Excellence (NICE) guidelines,^25^ patients at risk of infective endocarditis were not given antibiotic prophylaxis prior to the dental treatment, unless specifically recommended by their cardiologist. When recommended (in one patient from the CAD and five from the non-CAD group), antibiotic prophylaxis was administered according to the 2006 British Society of Antimicrobial Chemotherapy (BSAC) guidelines^26^ (3 g of oral amoxicillin administered 1 h prior to the procedure or 600 mg of oral clindamycin if the patient was allergic to penicillin).

### Sample collection and analysis

Non-fasting blood samples were obtained by a clean venipuncture from the antecubital fossa and with minimal stasis, before and 15 min and 24 h after the dental procedures. Plasma was immediately processed and stored in several aliquots at −70 °C for later analysis. Analysis performed at 48 h following dental extraction did not demonstrate any significant changes of most routine hematological and chemical blood tests.^27^

#### Biomarkers of myocardial injury

High-sensitivity cardiac troponin T (hs-TnT) was quantified according to the manufacturer’s protocol in a blind fashion, using electrochemiluminiscence immunoassay (Cobas, Roche Diagnostic, Mannheim, Germany) with a detection limit of 3.0 ng/L. Since the study involved patients undergoing simple extractions, the main aim was to check for any rise in hs-cTnT, as a link between dental procedures and acute cardiovascular events and not to demonstrate a rise above a certain level to diagnose myocardial infarction, which is not expected with such procedures.

#### Biomarkers of systemic inflammation

A broad panel of inflammatory biomarkers was measured by a multiplex high-sensitivity assay, including interleukin-1β (IL-1β), IL-6, IL-8, IL-10, IL-12, interferon-γ (IFN-γ), and tumor necrosis factor-α (TNF-α) (Meso Scale Discovery, Maryland, USA). Serum C-reactive protein (CRP) was measured by immunoturbidometry (Cobas, Roche Diagnostic, Mannheim, Germany).

#### Endotoxin activity

Lipopolysaccharide (LPS) endotoxin activity was measured by a zymogenic assay, according to the manufacturer's instructions (Endpoint Chromogenic Limulus Amebocyte Lysate, LAL Test, Lonza, Walkersville, Maryland, USA).

### Statistical analysis

All data are presented as mean and standard error of the mean unless specified. Demographic data at baseline were compared between groups at baseline (CAD and not CAD groups) by paired *t*-test for numerical variables and Fisher’s exact test for categorical variables. As no previous evidence was identified, the sample size calculation was based on a hypothetical difference in hs-cTn of 1 ng/ml between study groups (CAD and not CAD) after 24 h (SD of 1, power 90%), resulting in a *N* = 23 per group. Since this work represents a proof-of-concept study, no power calculation was performed. Because of the multiple variables to be analyzed and overcome the danger of obtaining spuriously significant results due to multiple testing, paired *t* tests were performed on all variables to compare different time points within each group. Only those variables that were found significant at the 0.05 level were analyzed using a hierarchical analysis of variance (ANOVA) with patients nested in groups and repeated measures over time. Post hoc Bonferroni comparisons were made to compare mean values at different time points in the ANOVA and a significance level of 0.025 was chosen instead of the conventional 0.05 to avoid spuriously significant results due to multiple testing. To assess the effect of any covariate that was distributed differently at baseline in the two groups, a regression analysis was performed with the outcome variable being the change in the variable of interest from one time point to the next and the explanatory variables being group, the significant demographic variable, and the interaction between the two. Following analysis of the primary marker (hs-TnT), the study sample was then categorized in two groups based on whether hs-TnT increased at 24 h or not. Data were graphically tested for normality, and logarithmic or square root transformations were made as needed before applying the adequate nonparametric tests. All analysis was performed with the statistical software package SPSS 21 (SPSS Inc., Chicago, IL, USA).

## Results

### Study patients at baseline (with and without CAD)

The CAD group comprised 28 patients (25 men and 3 women) with an age range of 41–91 years and the non-CAD group comprised 17 patients (11 men and 6 women) with an age range of 38–80 years. All patients attended the 24 -h visit follow-up and provided blood samples. There were no significant statistical differences between patients with and without CAD in terms of patient’s characteristics, associated diseases/risk factors, dental procedure-related variables, and medications used by the patients, except for antibiotic cover (3.6% in the CAD group and 29.4% in the non-CAD group, *p* = 0.023) (Table 1). The mean ± SEM values of biomarkers of all study patients and of patients with and without CAD at the three time points (pre, immediately post, and day 1 after treatment) are shown in Table 2.

**Table 1:** Summary of the clinical data of patients with and without CAD

Variable	CAD (*n* = 28)	Non-CAD (*n* = 17)	*p-*value
• *Patient’s characteristics*			
Male gender, *n* (%)	25 (89.3)	11 (64.7)	0.0626
Age, years (mean ± SD)	71.04 ± 10.54	65.94 ± 13.33	0.1906
• *Associated diseases/risk factors*			
Smoking, *n* (%)	2 (7.1)	3 (17.7)	0.3504
Hypertension, *n* (%)	18 (64.3)	10 (58.8)	0.7591
Diabetes, *n* (%)	11 (39.3)	2 (11.8)	0.0881
Chronic renal failure, *n* (%)	2 (7.1)	0 (0.0)	0.5192
COPD, *n* (%)	3 (10.7)	0 (0.0)	0.2788
BMI (mean ± SD)	29.61 ± 5.76	27.60 ± 5.57	0.4311
*Dental procedure-related variables*			
Number of extracted teeth (mean ± SD)	2.18 ± 1.36	2.12 ± 1.41	0.8876
Flap opened, *n* (%)	12 (42.8)	6 (35.3)	0.7566
Bone removal, *n* (%)	7 (25.0)	4 (23.5)	1.0000
Number of LA carpules (mean ± SD)	2.29 ± 0.88	2.21 ± 0.83	0.7612
Oral sedation (20 mg of diazepam), *n* (%)	10 (35.7)	4 (23.5)	0.5134
Preoperative GTN, *n* (%)	1 (3.6)	0 (0.0)	1.0000
Antibiotic cover, *n* (%)	1 (3.6)	5 (29.4)	0.0228
• *Medications*			
Single antiplatelets, *n* (%)	13 (46.4)	5 (29.4)	0.3523
Dual antiplatelets, *n* (%)	3 (10.7)	0 (0.0)	0.2788
Beta-blockers, *n* (%)	18 (64.3)	6 (35.3)	0.0727
Statins, *n* (%)	19 (67.9)	7 (41.2)	0.1206
Ezetimibe, *n* (%)	5 (17.6)	0 (0.0)	0.1401
Warfarin, *n* (%)	7 (25.0)	5 (29.4)	0.7428
ACE inhibitors, *n* (%)	16 (57.1)	7 (41.2)	0.3651
Calcium channel blockers, *n* (%)	7 (25.0)	3 (17.7)	0.7189
Nitrates, *n* (%)	4 (14.3)	0 (0.0)	0.2812
Cardiac glycosides, *n* (%)	4 (14.3)	0 (0.0)	0.2812
Proton-pump inhibitors, *n* (%)	5 (17.6)	2 (11.8)	0.6932
Diuretics, *n* (%)	8 (28.6)	7 (41.2)	0.5167
Alpha-1 blockers, *n* (%)	3 (10.7)	1 (5.9)	1.0000
ARBs, *n* (%)	1 (3.6)	2 (11.8)	0.5571
Oral hypoglycemics, *n* (%)	7 (25.0)	3 (17.7)	0.7189
Insulin, *n* (%)	1 (3.6)	0 (0.0)	1.0000
Seretide inhaler, *n* (%)	2 (7.1)	0 (0.0)	0.5192
Anticholinergics, *n* (%)	3 (10.7)	0 (0.0)	0.2788
Beta-2 agonists, *n* (%)	5 (17.6)	0 (0.0)	0.1401
Antiepileptics, *n* (%)	2 (7.1)	1 (5.9)	1.0000
Antidepressants, *n* (%)	4 (14.3)	1 (5.9)	0.6353

*ACE* *inhibitors*  angiotensin-converting enzyme inhibitors, *ARBS*  angiotensin receptor blockers, *BMI*   body mass index, *COPD*   chronic obstructive pulmonary disease, *GTN*   glyceryl trinitrate, *LA*   local anesthetic

**Table 2:** Mean levels of biomarkers of all study patients and of patients with and without CAD at the three time points (pre, post, and day 1)

Biomarker	Total patients^a^			CAD patients^b^			Non-CAD patients^c^		
	Pre	Post	Day 1	Pre	Post	Day 1	Pre	Post	Day 1
**•** *Biomarker of myocardial injury*									
hs-TnT (ng/L)	16.10 ± 2.42	15.28 ± 2.28	16.43 ± 2.45	17.91 ± 3.38	16.98 ± 3.22	18.34 ± 3.47	13.12 ± 3.18	12.47 ± 3.02	13.29 ± 3.23
**•** *Biomarkers of systemic inflammation*									
hs-CRP (mg/L)	4.16 ± 0.61	3.96 ± 0.59	9.69 ± 1.44	4.53 ± 0.86	4.38 ± 0.83	9.80 ± 1.85	3.56 ± 0.86	3.28 ± 0.80	9.51 ± 2.31
Fibrinogen (mg/l)	3.29 ± 0.49	3.22 ± 0.48	3.54 ± 0.53	3.20 ± 0.60	3.16 ± 0.60	3.43 ± 0.65	3.45 ± 0.84	3.35 ± 0.81	3.72 ± 0.90
IFN-γ (pg/ml)	2.36 ± 0.35	2.40 ± 0.36	7.59 ± 1.13	2.37 ± 0.45	2.46 ± 0.47	8.06 ± 1.52	2.34 ± 0.57	2.30 ± 0.56	6.82 ± 1.66
TNF-α (pg/ml)	10.52 ± 1.57	10.31 ± 1.54	11.37 ± 1.69	10.76 ± 2.03	10.40 ± 1.97	11.61 ± 2.19	10.12 ± 2,46	10.15 ± 2.46	10.99 ± 2.67
IL-1β (pg/ml)	0.44 ± 0.07	0.39 ± 0.06	0.47 ± 0.07	0.50 ± .0.09	0.39 ± 0.07	0.49 ± 0.09	0.33 ± 0.08	0.39 ± 0.09	0.44 ± 0.11
IL-6 (pg/ml)	1.96 ± 0.29	1.89 ± 0.28	3.92 ± 0.58	2.08 ± 0.39	1.99 ± 0.38	4.15 ± 0.78	1.77 ± 10.43	1.73 ± 0.18	3.54 ± 0.86
IL-8 (pg/ml)	4.25 ± 0.63	5.56 ± 0.83	6.51 ± 0.97	4.51 ± 0.85	5.23 ± 0.99	7.35 ± 1.39	3.83 ± 0.93	6.10 ± 2.09	5.13 ± 1.25
IL-10 (pg/ml)	7.87 ± 1.17	8.28 ± 1.23	8.52 ± 1.27	7.47 ± 1.41	8.07 ± 1.53	8.14 ± 1.54	8.53 ± 2.07	8.62 ± 2.09	9.15 ± 2.22
IL-12 (pg/ml)	7.33 ± 1.09	6.83 ± 1.02	7.45 ± 1.11	4.41 ± 0.83	4.25 ± 0.80	5.22 ± 0.99	12.14 ± 2.95	11.10 ± 2.69	11.13 ± 2.70
**•** *Biomarker of endotoxemia (LPS endotoxin)*									
LPS (LAL) (EU/ml)	1.02 ± 0.15	1.09 ± 0.16	1.41 ± 0.21	0.98 ± 0.19	1.05 ± 0.20	1.45 ± 0.22	1.08 ± 0.26	1.17 ± 0.28	1.34 ± 0.33

*IL* interleukin *IFN-γ* interferon-γ, *hs-TnT* highly sensitive troponin T, *hs-CRP* highly sensitive C-reactive protein, *LPS* lipopolysaccharide,*TNF-α* tumor necrosis factor-α

Values = mean ± SEM

^a^*n* = 45 for all except for fibrinogen = 43

^b^*n* = 28 for all except for fibrinogen = 27

^c^*n* = 17 for all except for fibrinogen = 16

#### Myocardial injury

**Fig. 1:**
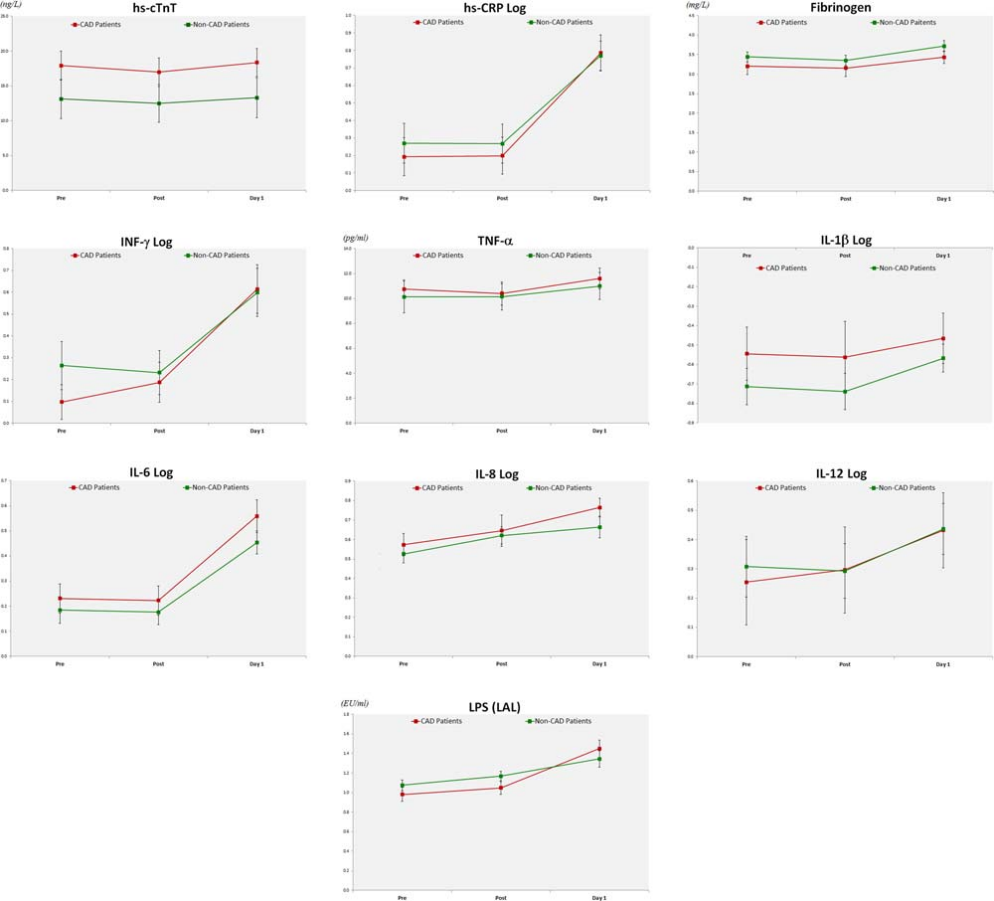
Changes in the estimated marginal means ± SEM in CAD patients (red line) and non-CAD patients (green line) of hs-cTnT, inflammation markers with statistically significant changes (hs-CRP log, fibrinogen, IFN-γ log, TNF-α, IL-1 log, IL-6 log, IL-8 log, and IL-12 log), and LPS endotoxin

Statistically significant changes (*p* < 0.05) were observed following dental procedures (Table 2) and ANOVA analysis showed significant difference (*p* < 0.025) between two or more of the time points for hs-TnT (Table 3). However, there was no statistical significant difference between the groups (CAD vs. no CAD) or in the interaction between the groups and the different time points, thus inferring that the change was similar for both groups (Table 3). Concentrations 24 h after the dental procedure were substantially higher than those obtained immediately post procedure (Table 3). Graphical studies demonstrated that hs-cTnT values were normally distributed and changes in the estimated marginal means are shown in Fig. 1.

**Table 3:** Hierarchical ANOVA and post hoc Bonferroni comparisons performed on hs-TnT, fibrinogen, TNF-α, LPS, and on hs-CRP, IFN-γ, IL-1β, IL-6, IL-8, and IL-12 after logarithmic transformation in patients with and without CAD

Biomarker	*P*-values between-subject effects using hierarchical ANOVA^a^			*P*-values between different time points using post hoc Bonferroni^b^		
	Group^c^	Time^d^	Group×time^e^	Pre–Post^f^	Pre-D1^g^	Post-D1^h^
**•** *Biomarker of myocardial injury*						
hs-TnT	0.227	0.025	0.805	0.118	>0.999	0.013
**•** *Biomarkers of systemic inflammation*						
hs-CRP log	0.741	<0.001	0.679	>0.999	<0.001	<0.001
Fibrinogen	0.270	0.006	0.835	>0.999	0.038	0.005
IFN-γ log	0.628	<0.001	0.418	>0.999	<0.001	<0.001
TNF-α	0.744	0.046	0.887	>0.999	0.137	0.040
IL-1β log	0.233	0.114	0.957	>0.999	0.352	0.180
IL-6 log	0.328	<0.001	0.672	>0.999	<0.001	<0.001
IL-8 log	0.422	<0.001	0.457	0.027	<0.001	0.011
IL-12 log	0.91	<0.001	0.579	>0.999	<0.001	<0.001
**•** *Biomarker of endotoxemia (LPS endotoxin)*						
LPS (LAL)	0.612	<0.001	0.151	0.633	<0.001	<0.001

*IL* interleukin, *IFN-γ* interferon-γ, *hs-TnT* highly sensitive troponin T, *hs-CRP* highly sensitive C-reactive protein, *LPS* lipopolysaccharide, *TNF-α* tumor necrosis factor-α

^a^Hierarchical ANOVA at a significance level of 0.025 performed on biomarkers that were found significant at the 0.05 level by paired t-test after logarithmic transformation

^b^Post hoc Bonferroni comparisons made to compare mean values at different time points of the ANOVA

at a significance level of 0.025 (significant difference shown in red)

^c^Group = comparing CAD vs. non-CAD patients

^d^Time = comparing the different time points (pretreatment, immediately post treatment, and day-1 treatment)

^e^Group × time = comparing the interaction between the groups and time points

^f^Pre–Post = comparing pretreatment and immediately post treatment

^g^Pre-D1 = comparing pre-treatment and day-1 treatment

^h^Post-D1 = comparing immediately post treatment and day-1 treatment

#### Systemic inflammation

Graphical studies of the biomarkers of systemic inflammation demonstrated that fibrinogen and TNF-α were normally distributed, while CRP, IFN-α, IL-1β, IL-6, IL-8, and IL-12 were not; thus, the latter required logarithmic transformation before further analysis using ANOVA. Changes in the estimated marginal means of these markers are shown in Fig. 1. Statistically significant changes (p < 0.05) were observed following dental procedures for hs-CRP, fibrinogen, IFN-γ, TNF-α, IL-1β, IL-6, IL-8, and IL-12 and no differences were noted at any of the time points for IL-10 (Table 2). Further analysis showed significant difference (<0.025) between two or more of the time points for hs-CRP, fibrinogen, IFN-γ, IL-6, IL-8, and IL-12 (Table 3), which were similar for both groups with and without CAD (Table 3). Concentrations observed at 24 h after the dental procedure were substantially higher than those obtained immediately after the procedure for all these biomarkers and higher than those obtained before the procedure for CRP, IFN-γ, IL-6, IL-8, and IL-12 (Table 3).

#### Endotoxemia

Statistical significant changes were observed following dental procedures for LPS (Table 2) and the kinetics of this biomarker was similar for both groups with and without CAD (Table 3). Concentrations were substantially higher 24 h after the dental procedure than those obtained immediately post procedure (Table 3). Graphical studies demonstrated that LPS endotoxin values were normally distributed and the changes in the estimated marginal means are being shown in Fig. 1.

As antibiotic cover was distributed differently at baseline in the two groups (Table 1), linear regression analysis, including antibiotic cover into the modeling, demonstrated a significant effect on the change of IL-6 between pretreatment and 24 h post treatment, as well as between immediately post treatment and 24 h post treatment, but this effect was not consistent for each study group. However, only one patient within the CAD group received antibiotic cover; therefore, detailed statistical analysis of this factor was not possible.

### Patients with and without hs-TnT rise

The hs-TnT rise group comprised 28 patients (22 men and 6 women) with an age range of 51–91 years and the hs-TnT non-rise group consisted of 17 patients (14 men and 3 women) with an age range of 38–86 years. There were no significant statistical differences between the two groups in terms of patient’s characteristics, associated diseases/risk factors, dental procedure-related variables, and medications used by the patients (Table 4).

**Table 4:** Summary of the clinical data of patients with and without hs-TnT rise

Variable	hs-TnT rise (*n* = 28)	hs-TnT non-rise (*n* = 17)	*p-*value
*• Patient’s characteristics*			
Male gender, *n* (%)	22 (78.6)	14 (82.3)	1.0000
Age, years (mean ± SD)	71.40 ± 9.24	65.35 ± 14.64	0.1400
*• Associated diseases/risk factors*			
Coronary artery disease	17 (60.7)	11 (64.7)	1.0000
Smoking, *n* (%)	3 (10.7)	2 (11.8)	1.0000
Hypertension	18 (64.3)	10 (58.8)	0.7591
Diabetes, *n* (%)	7 (25.0)	7 (41.2)	0.3262
Chronic renal failure, *n* (%)	1 (3.6)	1 (5.9)	1.0000
COPD, *n* (%)	2 (7.1)	1 (5.9)	1.0000
BMI (mean ± SD)	29.55 ± 5.70	27.93 ± 5.91	0.5209
*• Dental procedure-related variables*			
Number of extracted teeth (mean ± SD)	2.25 ± 1.38	2±1.37	0.5575
Flap opened, *n* (%)	13 (46.4)	5 (29.4)	0.3513
Bone removal, *n* (%)	7 (25.0)	4 (23.5)	1.0000
Numberof LA carpules (mean ± SD)	2.36 ± 0.9	2.09 ± 0.78	0.2935
Oral sedation (20 mg of diazepam), *n* (%)	7 (25.0)	7 (41.2)	0.3262
Preoperative GTN, *n* (%)	1 (3.6)	0 (0.0)	1.0000
Antibiotic cover, *n* (%)	5 (17.6)	1 (5.9)	0.3846
*• Medications*			
Single antiplatelets, *n* (%)	14 (50.0)	8 (47.1)	1.0000
Dual antiplatelets, *n* (%)	1 (3.6)	2 (11.8)	0.5471
Beta-blockers, *n* (%)	16 (57.1)	8 (47.1)	0.5521
Statins, *n* (%)	17 (60.7)	9 (52.9)	0.5754
Ezetimibe, *n* (%)	2 (7.1)	3 (17.7)	0.3590
Warfarin, *n* (%)	10 (35.7)	2 (11.8)	0.0962
ACE inhibitors, *n* (%)	15 (53.6)	8 (47.1)	0.7631
Calcium-channel blockers, *n* (%)	5 (17.6)	4 (23.5)	0.7109
Nitrates, *n* (%)	4 (14.3)	1 (5.9)	0.6343
Cardiac glycosides, *n* (%)	2 (7.1)	2 (11.8)	0.6262
Proton-pump inhibitors, *n* (%)	2 (7.1)	5 (29.4)	0.0858
Diuretics, *n* (%)	12 (42.9)	4 (23.5)	0.2187
ARBs, *n* (%)	2 (7.1)	1 (5.9)	1.0000
Oral hypoglycemics, *n* (%)	4 (14.3)	6 (35.3)	0.1434
Insulin, *n* (%)	1 (3.6)	0 (0.0)	1.0000
Seretide inhaler, *n* (%)	1 (3.6)	1 (5.9)	1.0000
Anticholinergics, *n* (%)	2 (7.1)	1 (5.9)	1.0000
Beta-2 agonists, *n* (%)	3 (10.7)	2 (11.8)	1.0000
Antiepileptics, *n* (%)	1 (3.6)	2 (11.8)	0.5471
Antidepressants, *n* (%)	3 (10.7)	2 (11.8)	1.0000

*ACE inhibitors*   angiotensin-converting enzyme inhibitors, *ARBS * angiotensin receptor blockers, *BMI *  body mass index, *COPD*   chronic obstructive pulmonary disease, *GTN*   glyceryl trinitrate, *LA*   local anesthetic

The mean ± SEM values of biomarkers of all study patients and of patients with and without hs-TnT rise at the three time points (pre, immediately post, and day 1 after treatment) are shown in Table 5.

**Table 5:** Mean levels of biomarkers of all study patients and of patients with and without hs-TnT rise at the three time points (pre, post, and day 1)

Biomarker	hs-TnT rise patients^a^			hs-TnT non-rise patients^b^		
	Pre	Post	Day 1	Pre	Post	Day 1
*• Biomarkers of systemic inflammation*						
hs-CRP (mg/L)	5.43 ± 1.03	5.14 ± 0.97	10.53 ± 1.99	2.07 ± 0.50	2.03 ± 0.49	8.31 ± 2.02
Fibrinogen (mg/l)	3.34 ± 0.63	3.29 ± 0.62	3.64 ± 0.69	3.22 ± 0.78	3.13 ± 0.76	3.38 ± 0.82
IFN-γ (pg/ml)	2.35 ± 0.44	2.38 ± 0.45	8.69 ± 1.64	2.37 ± 0.58	2.44 ± 0.59	5.78 ± 1.40
TNF-α (pg/ml)	10.97 ± 2.07	10.44 ± 1.97	12.15 ± 2.30	9.77 ± 2.37	10.09 ± 2.45	10.09 ± 2.45
IL-1β (pg/ml)	0.50 ± 0.09	0.42 ± 0.08	0.41 ± 0.08	0.33 ± 0.08	0.35 ± 0.08	0.57 ± 0.14
IL-6 (pg/ml)	2.02 ± 0.38	1.89 ± 0.36	3.92 ± 0.74	1.88 ± 0.46	1.89 ± 0.46	3.91 ± 0.95
IL-8 (pg/ml)	4.71 ± 0.89	5.37 ± 1.02	6.88 ± 1.30	3.50 ± 0.85	5.88 ± 1.43	5.90 ± 1.43
IL-10 (pg/ml)	6.63 ± 1.25	6.65 ± 1.26	7.23 ± 1.37	9.91 ± 2.41	10.97 ± 2.66	10.66 ± 2.59
IL-12 (pg/ml)	4.30 ± 0.81	4.02 ± 0.76	5.03 ± 0.95	12.33 ± 2.99	11.46 ± 2.78	11.45 ± 2.78
*• Biomarker of endotoxemia (LPS endotoxin)*						
LPS (LAL) (EU/ml)	0.98 ± 0.19	1.09 ± 0.21	1.44 ± 0.27	1.08 ± 0.26	1.10 ± 0.27	1.36 ± 0.33

*IL* interleukin, *IFN-γ* interferon-γ, *hs-Tn*T highly sensitive troponin T, *hs-CRP* highly sensitive C-reactive protein, *LPS* lipopolysaccharide, *TNF-α* tumor necrosis factor-α

Values *=* mean ± SEM

^a^*n* *=* 28 for all except for fibrinogen = 27

^b^*n* *=* 17 for all except for fibrinogen = 16

#### Systemic inflammation

**Fig. 2:**
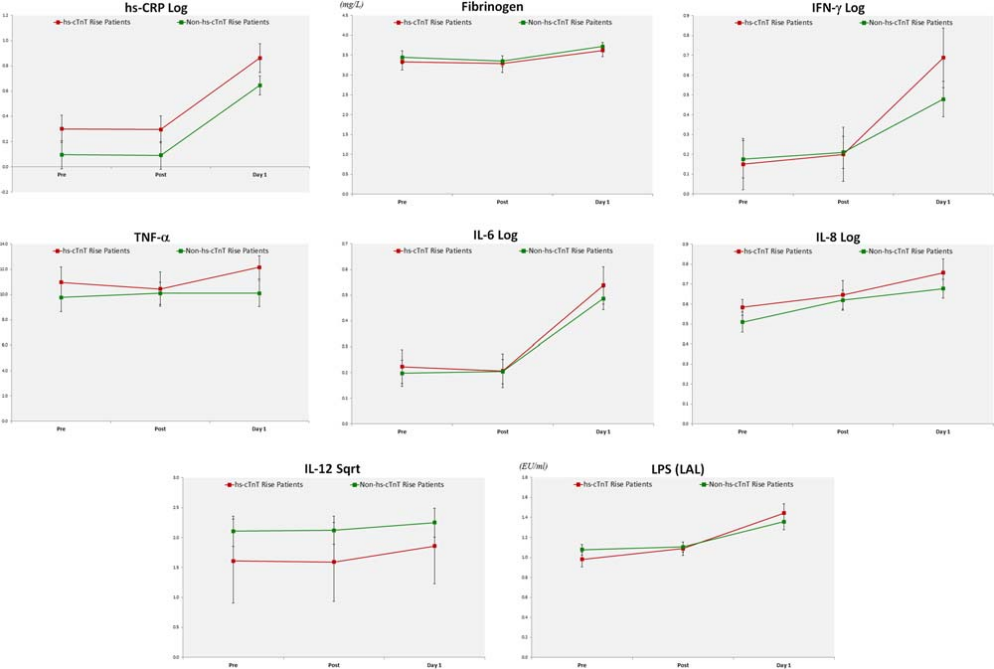
Changes in the estimated marginal means ± SEM in patients with hs-cTnT rise (red line) and patients without hs-cTnT (green line) of inflammation markers with statistically significant changes (hs-CRP log, fibrinogen, IFN-γ log, TNF-α, IL-6 log and IL-8 log, and IL-12 Sqrt) and LPS endotoxin

Statistically significant changes were observed following dental procedures in both groups with and without hs-cTnT rise for hs-CRP, fibrinogen, IFN-γ, TNF-α, IL-6, IL-8, and IL-12, and no significant differences were noted for IL-1β and IL-10 (Table 6). Graphical studies of these biomarkers demonstrated that fibrinogen and TNF-α were normally distributed, while CRP, IFN-γ, IL-6, IL-8, and IL-12 were not, and thus required logarithmic (for CRP, IFN-γ, IL-6, and IL-8) and square root (for IL-12) transformation before further analysis using ANOVA. Changes in the estimated marginal means ± SEM of these markers are shown in Fig. 2.

**Table 6:** Hierarchical ANOVA and post hoc Bonferroni comparisons performed on hs-TnT, fibrinogen, TNF-α, LPS, and on hs-CRP, IFN-γ, IL-1β, IL-6, IL-8 after logarithmic transformation, and IL-12 after square root transformation, in patients with and without hs-TnT rise

Biomarker	*P*-values between-subject effects using hierarchical ANOVA^a^			*P*-values between different time points using post hoc Bonferroni^b^		
	Group^c^	Time^d^	Group × time^e^	Pre–Post^f^	Pre-D1^g^	Post-D1^h^
*• Biomarkers of systemic inflammation*						
hs-CRP log	0.139	<0.001	0.994	>0.999	<0.001	<0.001
Fibrinogen	0.352	0.012	0.812	>0.999	0.037	0.005
IFN-γ log	0.667	<0.001	0.176	>0.999	<0.001	<0.001
TNF-α	0.434	0.096	0.138	>0.999	0.123	0.034
IL-6 log	0.697	<0.001	0.811	>0.999	<0.001	<0.001
IL-8 log	0.404	<0.001	0.652	0.028	<0.001	0.011
IL-12 sqrt	0.434	<0.001	0.218	>0.999	<0.001	<0.001
*• Biomarker of endotoxemia (LPS endotoxin)*						
LPS(LAL)	0.888	<0.001	0.356	0.647	<0.001	<0.001

*IL* interleukin, *IFN-γ* interferon-γ, *hs-TnT* highly sensitive troponin T, *hs-CRP* highly sensitive C-reactive protein, *LPS* lipopolysaccharide, *TNF-α* tumor necrosis factor-α

^a^Hierarchical ANOVA at a significance level of 0.025 performed on biomarkers that were found significant at the 0.05 level by paired t-test after logarithmic or square root transformation

^b^Post hoc Bonferroni comparisons made to compare mean values at different time points of the ANOVA

at a significance level of 0.025 (significant difference shown in red)

^c^Group = comparing hs-TnT rise vs. non-rise patients

^d^Time = comparing the different time points (pretreatment, immediately post treatment, and day-1 treatment)

^e^Group × time = comparing the interaction between the groups and time points

^f^Pre–Post = comparing pretreatment and immediately post treatment

^g^Pre-D1 = comparing pretreatment and day-1 treatment

^h^Post-D1 = comparing immediately post treatment and day-1 treatment

#### Endotoxemia

Statistically significant changes for LPS were observed in both groups following dental procedures (Table 6). Graphical studies demonstrated that LPS endotoxin values were normally distributed and changes in the estimated marginal means ± SEM are shown in Fig. 2.

## Discussion

This study confirmed that dental surgery is associated with a rise in hs-TnT, bacterial LPS endotoxin, and acute systemic inflammation. These changes were similar in both patients with and without CAD, although the changes in systemic inflammation and endotoxemia were more pronounced in those with a rise in hs-TnT. These findings were independent of age, gender, smoking, and other traditional cardiovascular risk factors.

### Myocardial injury

This study demonstrated for the first time statistically significant elevations of hs-TnT within 24 h following dental extractions, irrespective of whether patients had CAD or not. A previous investigation employing a conventional cTnT assay did not find a rise in cTnT levels following dental extractions.^28^ The high-sensitivity assay used in this study may represent the most plausible explanation for the differences with our findings.^29^ While measurable, the presently observed minute increases in hs-TnT probably reflect a transitory impairment of myocardial integrity. This might be due to either increased cellular permeability and early troponin release from the cytosolic pool during ischemia (similar to those reported following strenuous exercise)^30^ or the consequence of breakdown of myocytes following microthrombosis (similar to that occurring following coronary interventions)^31,32^ or a combination of both mechanisms.^33^ During cardiac ischemia, cardiac myocytes develop blebs on the surface of their plasma membrane and release cytoplasmic contents, without undergoing necrosis.^34^ Blebs have been defined as “bubbles” developing from the plasma membrane in response to temporary ischemia, which can either be resorbed or shed into the circulation when reoxygenation later occurs. However, when the ischemia is severe and prolonged (as in ACS), the blebs grow and collapse, and cell necrosis occurs.^34^ Reversible ischemia-induced bleb release is characterized by a brief peak, and a rapid decrease and normalization within 72 h,^35^ while irreversible myocyte necrosis release is characterized by a steep increase and prolonged elevation for at least 4–7 days.^36^ Hence, hs-TnT measurement 4–7 days after the procedure could differentiate between the two mechanisms. Alternatively, using delayed enhancement magnetic resonance imaging,^37^ myocardial microinfarction could be verified.^38^

The clinical significance of the present observations in patients with CAD remains to be established. Indeed, peri-procedural minor myocardial injury detected by cardiac troponin following coronary interventions is associated with a worsened cardiac outcome of CAD patients.^2,3^ If proven of clinical significance, the protocols for even minor invasive dental treatment in patients with CAD would need to be revisited. Further support for the present findings comes from the epidemiologic evidence of a modest but statistically significant increase of acute vascular events following invasive dental procedures in a large US sample^9^ and a recent observation of a case-series report of major adverse outcomes associated with planned exodontia before cardiac operations.^5^

Then again, a recent study reported that Medicare beneficiaries who underwent dental procedures within 30–180 days after an ischemic vascular event were not at an increased risk of experiencing a second event.^39^ However, this report was based on a combined survey and administrative data set and did not contain clinical or laboratory diagnostic information. In addition, the survey recorded many noninvasive dental procedures, including radiographs, examinations, restorations, crowns, bridges and complete and removable dentures, orthodontics, and other services, which are not expected to trigger vascular injury.^39^

### Systemic inflammation

Intensive periodontal treatment (including dental extractions) leads to transient impaired brachial artery flow-mediated dilation (a measure of endothelial function) and increased markers of inflammation and endothelial activation in the first 30 days after treatment and then followed by a progressive improvement 6 months after baseline.^5,7^ The more invasive the dental treatment, the greater the rise in inflammation markers.^8^ In this investigation, a large array of inflammatory biomarkers increased following dental treatment, confirming that a simple dental extraction can trigger a systemic inflammatory response, possibly due to endotoxemia and local tissue injury. The rise in hs-TnT in the same patients might support the involvement of systemic inflammatory response in the etiology of ischemia/injury observed following coronary interventions in CAD patients,^31,32^ which is possibly caused by the induction of thrombogenesis and/or microembolization within the myocardium, leading to microinfarcts.

### Bacterial burden

Bacteremia following invasive dental procedures or simple tooth brushing or even chewing has been previously established.^40–47^ Collagen-like molecule on the surface of *Streptococcus sanguis* and *Porphyromonas gingivalis* can induce a hypercoagulable state by increasing serum fibrinogen, white blood cell number, and platelet aggregation.^10–12^ The results of this study confirm a raised endotoxin activity 24 h following dental extraction, which might explain the induction of a hypercoagulable state and suggest either a direct pathogen effect at sites distant from the oral cavity or host inflammatory/immune response.^48,49^ Low levels of endotoxin induce inflammatory responses in human monocytes and macrophages, and vascular smooth muscle cells and intact human blood vessels also exhibit a profound cytokine release, superoxide production, and monocyte adhesion in response to exodontia.^15,16^ In addition, LPS can significantly decrease fibrous cap thickness and increase macrophage and lipid contents of atherosclerotic plaques, which can lead to their disruption.^49^ In vivo studies have demonstrated that increased LPS endotoxin levels due to infection or sepsis can cause apoptosis through the activation of caspases in myocardial cells via the induction of proinflammatory acute-phase proteins and cytokines within these cells, resulting in contractile dysfunction and sarcomeric destruction that are reflected in an increase in peripheral blood troponin levels.^37,38^ The significantly increased levels of LPS endotoxin in these patients might be associated with similar cellular events, which may provide another or additional mechanism by which hs-TnT levels increase following dental extraction.

**Fig. 3:**
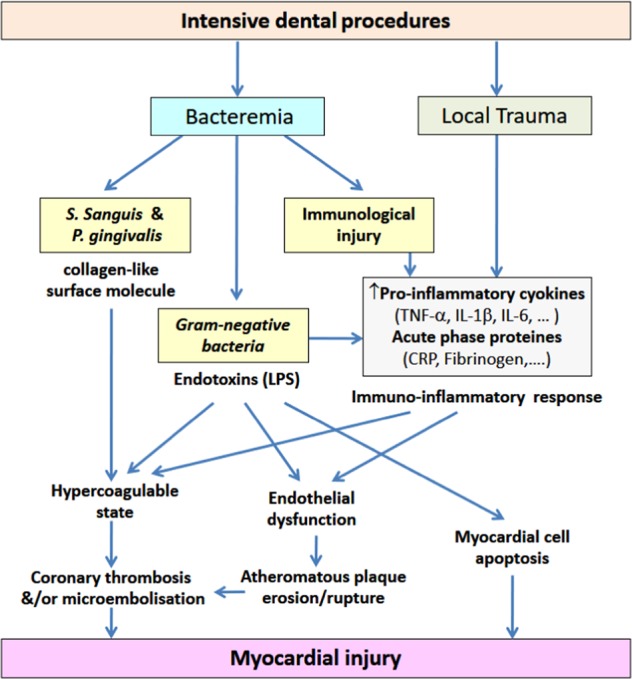
Possible mechanisms of myocardial injury following dental extraction. CRP, C-reactive protein; IL, interleukin; ICAM, intercellular adhesion molecule; LPS, lipopolysaccharide; *P. gingivalis*, *Porphyromonas gingivalis*; *S. sanguis*, *Streptococcus sanguis*; TNF-α, tumor necrosis factor-α; VCAM, vascular cell adhesion molecule

In summary, this study demonstrated that dental surgery is associated with potential minor myocardial injury in the form of increased hs-TnT levels. It also confirmed that exodontia is also associated with a rise in bacterial LPS endotoxin and acute systemic inflammation. These changes were similar in both patients with and without CAD, although the changes in systemic inflammation and endotoxemia were more pronounced in those with a rise in hs-TnT. As consequences of bacteremia and local trauma and tissue injury, these changes suggest a possible connection between these mechanisms and minor myocardial injury following dental surgery as summarized in Fig. 3.

Several limitations should be considered in this study. First, most of the studied patients were older males (mean age of 69 ± 12 years) with some form of vascular diseases. Indeed, the non-CAD patients may still have a non-obstructive CAD with a considerable degree of inflammatory atherosclerosis, which possibly explains the lack of significant difference between the groups. Future studies should recruit more patients and include a greater proportion of females, younger subjects, and healthy controls (disease free). Second, the presence of several confounding factors, such as sedation, extent of dental extraction, use of antibiotic prophylaxis, and the use of preoperative GTN could have influenced our results. These factors could have masked an expected significant difference between the groups and between the time points of the biomarkers examined. Such confounding factors should be taken into account in future studies. Third, the study did not include early samples following dental extraction, resulting in a lack of evidence of an early rise of various inflammatory markers (such as IL-1β, TNF-α, and P-selectin). Future studies should thus include earlier sampling methods to characterize the kinetics of the immediate (6–12-h) host response. An additional limitation is the lack of any microbial (bacterial) serum/plasma quantification to assess whether the alterations seen were also correlated with the level of circulating bacterial endotoxins (which represent mainly the gut rather than oral microflora activity). Nevertheless, this is the first study to propose a number of plausible mechanisms, including minor myocardial injury, as assessed by increased hs-TnT levels as a possible link between invasive dental treatment and acute cardiovascular events.

## Conclusion

Simple dental extractions were associated with potential minor myocardial injury in the form of increased hs-TnT levels in both patients with and without CAD, representing a possible link between invasive dental treatment and increased risk of acute cardiovascular events. Exodontia was also associated with acute systemic inflammation and endotoxemia, independent of age, gender, associated diseases and risk factors, dental procedure, or medication differences. These changes were more evident in those patients with hs-TnT rise. If replicated in larger studies, the present observations indicate that invasive dental treatment (as simple as a single dental extraction) could negatively affect upon the myocardial health of dental patients, especially those with CAD.
